# Supplementary material for: Measured and Estimated Glomerular Filtration Rate to Evaluate Rapid Progression and Changes over Time in Autosomal Polycystic Kidney Disease: Potential Impact on Therapeutic Decision-Making
Source: Int J Mol Sci. 2024 May 5;25(9):5036. doi: 10.3390/ijms25095036 (PMC11084593; doi:10.3390/ijms25095036)
Supplement: Supplementary file 1 [file ijms-25-05036-s001.zip › ijms-2982794-supplementary.pdf]

Supplementary material for Article:

Measured and estimated GFR to evaluate rapid progression and GFR changes over time in ADPKD: potential impact on therapeutic decision-making.

Authors:

Rosa Miquel-Rodríguez [1], Beatriz González-Toledo [2], María-Vanesa Pérez-Gómez [2,3,4], Marian Cobo [1], Patricia Delgado-Mallén [1], Sara Estupiñán [1], Coriolano Cruz-Perera [5], Laura Díaz-Martín [5], Federico González Rinne [5], Alejandra González-Delgado [6], Armando Torres [1,5,7], Flavio Gaspari [5], Domingo Hernández-Marrero [1,5,7], Alberto Ortiz [2,3,4], Esteban Porrini[5,7], Sergio Luis Lima [5, 6, 7]

1 Nephrology Department, Complejo Hospitalario Universitario de Canarias, La Laguna, Spain.
2 Department of Nephrology and Hypertension, IIS-Fundacion Jimenez Diaz UAM, Madrid, Spain.
3 Department of Medicine, RICORS2040, Madrid, Spain.
4 Departamento de Medicina, Facultad de Medicina, Universidad Autónoma de Madrid, Madrid, Spain.
5 Laboratory of Renal Function (LFR), Faculty of Medicine, Complejo Hospitalario Universitario de Canarias, University of La Laguna, La Laguna, Spain.
6 Department of Laboratory Medicine, Complejo Hospitalario Universitario de Canarias, Tenerife, Spain.
7 Instituto de Tecnologías Biomédicas (ITB), Faculty of Medicine, University of La Laguna, La Laguna, Spain

Contents:

Table S1 on page 2
Table S2 on page 6

Table S1: mGFR and eGFR decline (creatinine-based) in all cases included for analysis grouped in patients with rapid progression, moderate progression, stable or improvement in GFR over time.
Table S2: mGFR and eGFR decline (cystatin-c-based) in all cases included for analysis grouped in patients with rapid progression, moderate progression, stable or improvement in GFR over time.

Table S1: mGFR and eGFR decline (creatinine-based) in all cases included for analysis grouped in patients with rapid progression, moderate progression, stable or improvement in GFR over time.

		Creatinine-based formulas							
Patient	mGFR	Effersoe	CG	aMDRD	MCQ	CKD-EPI	LMRev	FAS	EKFC
99	-35.2	-28.9	-34.4	-32.4	-45.7	-39.2	-36.2	-34.2	-36.6
135	-27.0	-32.6	-46.2	-33.5	-46.7	-37.6	-34.5	-39.0	-37.9
134	-17.6	-9.0	-18.1	-9.8	-5.8	-11.7	-8.9	-10.7	-11.2
20	-16.4	1.9	-0.7	1.7	1.0	2.4	2.4	2.1	3.2
64	-12.1	-13.8	-25.3	-14.4	-23.8	-16.7	-15.3	-16.9	-16.8
15	-11.2	-4.6	-8.3	-5.2	-8.6	-6.1	-6.7	-5.4	-5.7
2	-10.3	-5.2	-10.3	-5.7	-9.3	-6.6	-6.5	-6.3	-6.8
9	-10.2	-5.5	-6.8	-6.5	-12.2	-7.8	-6.8	-6.2	-6.9
8	-8.6	-6.6	-6.2	-7.0	-10.6	-8.2	-6.5	-7.6	-8.0
75	-8.2	-2.1	-2.6	-2.7	-0.1	-1.8	-1.8	-2.5	-1.9
107	-8.2	-7.0	-9.5	-7.6	-11.5	-8.7	-8.2	-8.4	-8.9
5	-7.4	-5.6	-8.0	-5.4	-5.7	-6.1	-5.2	-7.1	-6.4
18	-7.4	2.9	-1.9	2.8	1.2	1.9	0.9	2.7	0.8
113	-7.4	-3.2	-4.4	-3.4	-5.0	-3.9	-4.4	-3.9	-4.0
39	-7.3	-4.5	-6.0	-4.6	-6.2	-5.2	-4.3	-5.4	-5.3
111	-7.2	-7.3	-11.6	-7.3	-8.0	-8.1	-6.1	-8.9	-8.3
42	-7.0	-5.3	-7.1	-5.3	-7.5	-6.1	-4.6	-6.6	-6.3
21	-6.8	-2.1	-2.8	-2.2	-4.0	-2.6	-2.5	-2.6	-2.7
84	-6.7	-4.7	-7.8	-5.8	-10.4	-7.1	-6.6	-5.6	-6.0
87	-6.5	-7.9	-7.4	-7.6	-11.4	-8.0	-8.3	-8.8	-7.4
105	-6.5	-2.1	-2.1	-2.3	-3.3	-2.6	-1.9	-2.5	-2.7
51	-6.3	1.8	1.1	1.1	-1.0	-0.1	1.4	2.0	-0.5
25	-6.2	-2.9	-3.5	-3.3	-5.4	-3.9	-4.3	-3.4	-3.5
6	-6.0	-2.8	-2.8	-2.7	-2.5	-2.8	-2.2	-3.4	-2.8
16	-6.0	-3.1	-3.6	-3.8	-6.8	-4.6	-4.3	-3.6	-3.9
66	-5.9	-2.8	-3.6	-2.9	-5.3	-3.4	-3.4	-3.4	-3.3
11	-5.7	-2.4	-3.5	-3.1	-1.4	-4.0	-2.7	-2.8	-3.3
85	-5.7	-4.7	-12.2	-5.8	-2.4	-6.8	-4.7	-5.7	-6.3
28	-5.4	-3.9	-3.2	-4.5	-5.3	-5.6	-4.5	-4.6	-5.3
140	-5.3	-18.3	-19.0	-17.9	-24.5	-19.5	-17.5	-21.3	-18.9
3	-5.0	-3.6	-4.7	-3.6	-3.7	-4.0	-3.1	-4.5	-4.2
13	-5.0	-0.6	-0.9	-0.7	-1.2	-1.0	-0.7	-0.7	-1.1
38	-5.0	-5.7	-6.8	-5.8	-10.1	-6.6	-6.2	-6.4	-6.3
73	-4.9	-3.0	-2.8	-3.2	-6.3	-3.8	-4.4	-3.5	-3.6
129	-4.9	-0.6	-2.5	-0.9	-0.6	-0.6	-0.9	-0.9	-0.9
93	-4.8	-1.6	-2.7	-1.8	-3.2	-2.3	-2.1	-2.0	-2.4
98	-4.8	-2.9	-3.2	-2.7	-2.6	-2.7	-2.1	-3.4	-2.7
52	-4.7	-4.3	-5.1	-4.3	-6.1	-4.7	-4.3	-5.0	-4.7
81	-4.6	-2.5	-2.7	-4.2	-5.2	-4.7	-1.5	-2.8	0.1
82	-4.5	-2.2	-2.9	-2.4	-4.7	-3.0	-3.1	-2.6	-2.9

19	-4.4	1.8	0.6	1.5	1.1	1.7	0.6	1.7	1.3
94	-4.4	-1.2	-4.3	-2.1	-2.8	-2.7	-1.5	-1.4	-1.6
26	-4.3	-7.4	-8.1	-8.9	-9.9	-9.3	-6.2	-8.1	-7.6
91	-4.3	-1.7	-3.4	-2.8	-2.7	-1.8	-1.5	-1.9	-0.6
71	-4.2	-0.8	-1.0	-1.5	-1.3	-1.9	-0.9	-1.0	-1.1
97	-4.2	-4.3	-5.5	-4.6	-8.8	-5.5	-6.1	-5.2	-5.4
110	-4.2	-5.4	-8.1	-5.2	-7.7	-5.8	-4.5	-6.6	-5.7
58	-4.1	-1.4	-5.2	-1.9	-2.9	-2.4	-1.9	-1.7	-1.7
122	-4.1	-6.1	-13.9	-7.5	-11.0	-8.9	-6.2	-7.0	-7.5
86	-3.9	-3.9	-3.8	-4.1	-6.9	-4.7	-4.4	-4.5	-4.6
95	-3.9	3.4	4.3	3.2	0.7	1.3	1.8	3.6	1.0
117	-3.9	-9.3	-9.5	-8.8	-9.7	-9.3	-6.8	-10.9	-9.0
125	-3.9	-0.8	-2.1	-1.2	-1.2	-1.7	-1.2	-1.0	-1.8
12	-3.8	-2.0	-4.5	-2.3	-2.8	-2.8	-2.5	-2.6	-2.9
109	-3.8	-7.2	-10.4	-7.2	-10.6	-8.0	-8.2	-8.5	-7.8
60	-3.7	-2.0	-3.4	-2.9	-5.0	-3.5	-2.3	-2.2	-2.6
27	-3.6	-0.3	-0.1	-0.7	-1.6	-1.0	-0.7	-0.3	-0.4
40	-3.6	-4.0	-4.7	-4.2	-5.1	-4.7	-3.5	-4.9	-4.9
53	-3.6	-5.6	-8.1	-6.7	-9.8	-8.1	-5.4	-6.3	-7.0
70	-3.5	-3.1	-4.4	-3.5	-5.8	-4.1	-4.4	-3.7	-4.2
124	-3.4	-4.9	-5.7	-4.7	-5.0	-5.0	-3.9	-5.9	-5.0
133	-3.4	-3.0	-5.1	-2.9	-3.4	-3.2	-2.7	-3.7	-3.3
24	-3.3	0.6	-0.3	0.5	0.9	0.4	0.5	0.6	0.2
36	-3.3	-4.5	-9.5	-4.5	-6.5	-5.1	-5.3	-5.4	-5.1
103	-3.1	-4.2	-4.6	-4.2	-7.8	-4.7	-5.4	-4.8	-4.4
119	-3.0	0.4	-0.3	0.3	0.3	0.1	0.2	0.3	-0.1
43	-2.9	-2.9	-3.9	-3.2	-2.7	-3.9	-3.2	-3.4	-3.7
50	-2.9	-3.0	-4.0	-3.0	-4.4	-3.4	-2.6	-3.7	-3.4
68	-2.9	-1.3	-2.0	-2.1	-1.5	-2.5	-1.4	-1.5	-1.7
114	-2.8	-1.2	0.1	-1.7	-0.4	-2.3	-1.6	-1.5	-2.3
56	-2.6	-2.9	-3.3	-2.8	-3.8	-3.2	-2.4	-3.6	-3.2
101	-2.6	-8.3	-11.6	-11.8	-1.7	-4.8	-4.5	-9.1	-2.2
29	-2.5	-1.7	-2.5	-1.9	-3.1	-2.3	-2.7	-2.1	-2.4
80	-2.5	-2.4	-1.8	-2.8	-2.6	-3.5	-2.8	-2.9	-3.4
96	-2.5	-4.1	-3.9	-5.9	-0.9	-7.3	-3.7	-4.6	-5.4
55	-2.4	-1.0	-2.0	-1.7	-3.0	-2.0	-1.4	-1.1	-1.3
67	-2.4	-6.0	-13.5	-7.7	-2.8	-4.5	-4.9	-7.0	-3.4
61	-2.3	1.5	1.3	1.3	0.1	1.2	0.8	1.6	0.9
65	-2.3	0.3	0.7	-0.1	0.6	-0.4	-0.1	0.3	-0.5
139	-2.3	-3.4	-6.7	-5.0	-7.6	-5.6	-3.0	-3.9	-1.5
123	-2.1	3.6	2.0	3.8	-1.4	0.0	1.8	4.1	0.7
45	-2.0	-3.1	-3.1	-2.9	-3.5	-3.1	-2.3	-3.6	-3.0
74	-2.0	-1.9	-2.3	-1.9	-3.7	-2.3	-2.6	-2.2	-2.2
108	-2.0	-0.6	-0.7	-0.8	-0.2	-1.3	-1.0	-0.8	-1.3
138	-1.9	-2.3	-2.5	-3.8	1.1	-0.4	-0.1	-2.6	-0.4
1	-1.8	-6.5	-8.6	-7.1	-12.8	-8.4	-8.0	-7.6	-8.4

4	-1.8	-1.8	-3.2	-2.0	-2.8	-2.3	-1.7	-2.2	-2.5
34	-1.7	-3.2	-3.6	-3.3	-6.1	-3.8	-4.2	-3.8	-3.7
23	-1.6	-1.0	-2.7	-1.4	-2.2	-1.8	-1.5	-1.4	-2.0
63	-1.6	-1.6	-2.4	-1.8	-2.7	-2.3	-2.1	-1.9	-2.3
131	-1.6	0.2	-0.7	0.1	-1.5	-0.1	-0.5	0.0	-0.4
46	-1.5	-1.6	-2.9	-1.6	-2.5	-1.8	-1.5	-2.0	-1.9
54	-1.5	0.1	-1.6	-0.1	-0.3	-0.3	-0.4	-0.1	-0.5
116	-1.4	-2.6	-3.2	-3.3	-0.7	-4.2	-2.8	-3.2	-4.2
92	-1.3	-1.5	-1.6	-1.6	-2.3	-1.8	-1.6	-1.8	-1.8
47	-1.2	-1.9	-2.0	-1.9	-2.2	-2.1	-1.6	-2.3	-2.2
83	-1.1	-1.1	-0.7	-1.5	-1.9	-2.1	-1.6	-1.4	-2.2
78	-1.0	-4.4	-5.0	-5.1	-9.1	-6.2	-4.9	-5.1	-6.2
30	-0.8	-2.3	-5.0	-2.1	-2.6	-2.3	-2.0	-2.9	-2.4
104	-0.6	0.0	-0.5	-0.2	-0.3	-0.3	-0.2	-0.1	-0.4
121	-0.6	2.0	1.3	1.9	3.4	2.0	2.0	2.2	1.6
14	-0.5	-10.2	-11.9	-11.2	-19.8	-13.1	-14.1	-11.9	-13.1
49	-0.4	-1.9	-3.2	-2.5	-4.1	-3.1	-2.3	-2.4	-3.3
37	-0.3	2.0	2.0	1.9	0.5	2.1	1.6	2.2	1.7
112	-0.3	-6.7	-10.2	-9.2	-1.1	-5.4	-4.9	-7.7	-3.8
118	-0.3	-3.5	-3.2	-3.3	-4.5	-3.4	-3.4	-3.9	-3.2
88	-0.2	-1.9	-2.6	-2.0	-3.4	-2.3	-1.8	-2.3	-2.3
90	-0.2	1.4	1.5	1.2	0.6	1.1	0.8	1.5	0.8
72	-0.1	-3.3	-4.3	-3.8	-5.4	-4.7	-4.1	-4.0	-4.6
33	0.1	-14.2	-12.6	-15.1	-0.6	-5.3	-8.2	-15.6	-4.9
62	0.1	0.5	3.4	-0.2	2.2	-0.5	0.6	0.7	0.4
100	0.1	-1.2	-1.9	-1.4	-1.8	-1.6	-1.2	-1.5	-1.7
136	0.1	-2.0	-2.0	-2.0	-3.5	-2.3	-1.4	-2.3	-2.2
7	0.2	-11.9	-17.6	-14.8	-3.0	-13.0	-10.2	-13.7	-10.9
10	0.2	-4.0	-4.8	-4.9	-2.1	-5.3	-4.0	-4.9	-5.3
48	0.2	-3.2	-3.7	-3.9	-5.8	-4.8	-4.1	-3.9	-4.7
59	0.2	-2.3	-4.5	-3.1	-0.3	-4.0	-2.2	-2.7	-3.0
77	0.2	-0.2	-0.1	-1.1	-0.3	-1.2	0.1	-0.2	-0.1
17	0.4	-4.9	-4.8	-6.1	-1.4	-7.7	-4.8	-5.6	-6.3
41	0.7	-3.3	-3.6	-3.9	-2.9	-3.3	-2.8	-3.7	-3.2
76	0.7	0.1	0.2	-0.6	-0.6	-0.6	0.1	0.1	0.1
32	0.9	-14.2	-14.2	-14.1	-23.2	-15.4	-13.6	-15.6	-14.1
22	1.1	0.9	0.1	0.8	1.1	0.7	0.8	0.9	0.5
69	1.2	-2.1	-4.7	-2.6	-4.5	-3.2	-2.6	-2.6	-3.4
79	1.2	-1.2	-1.8	-2.2	-1.0	-2.7	-1.3	-1.4	-1.6
44	1.4	3.1	7.0	3.0	-0.1	1.2	2.0	3.6	1.8
89	2.0	0.1	-0.8	-1.1	-0.4	-0.4	0.8	0.1	1.5
35	2.3	1.3	2.1	0.9	0.4	1.2	1.5	1.5	1.2
106	2.5	0.6	0.8	0.2	0.0	0.0	0.1	0.5	-0.3
115	2.5	-3.9	-5.8	-5.2	-1.1	-6.4	-3.6	-4.5	-5.0
130	2.5	0.5	-4.0	-0.3	-3.4	-2.5	0.3	0.4	-1.0
120	2.8	-3.5	-3.8	-4.9	0.6	-6.1	-2.9	-3.9	-4.6

127	2.9	3.2	4.1	2.9	4.5	3.7	3.8	3.8	4.3
132	3.0	16.8	23.0	18.4	10.8	22.2	16.1	19.5	20.7
102	3.2	3.5	9.9	3.3	4.8	3.8	4.0	4.4	4.2
128	3.6	-8.2	-10.1	-10.9	0.0	-6.0	-6.1	-9.4	-4.4
126	7.0	3.4	12.6	2.9	5.5	3.4	4.6	4.2	3.8
57	7.3	2.7	5.3	2.4	0.5	2.3	2.3	3.2	2.9
137	7.9	0.2	0.3	-0.6	-1.6	-0.3	0.7	0.3	1.3
31	8.6	-1.0	-2.1	-1.2	-2.2	-1.6	-1.5	-1.3	-1.7
**Counts**									
**≤-3**	66	61	78	66	66	74	60	68	69
**>-3 & <-1**	31	39	28	41	35	33	43	35	35
**≥-1 & <1**	25	23	19	17	28	18	24	20	22
**≥1**	18	17	15	16	11	15	13	17	14
**%**									
**≤-3**	47.1%	43.6%	55.7%	47.1%	47.1%	52.9%	42.9%	48.6%	49.3%
**>-3 & <-1**	22.1%	27.9%	20.0%	29.3%	25.0%	23.6%	30.7%	25.0%	25.0%
**≥-1 & <1**	17.9%	16.4%	13.6%	12.1%	20.0%	12.9%	17.1%	14.3%	15.7%
**≥1**	12.9%	12.1%	10.7%	11.4%	7.9%	10.7%	9.3%	12.1%	10.0%

Table S2: mGFR and eGFR decline (cystatin-c-based) in all cases included for analysis grouped in patients with rapid progression, moderate progression, stable or improvement in GFR over time.

		Cystatin-C formulas				Cystatin-C & Creatinine formulas			
Patient	mGFR	LeBricon	CKD-EPI	FAS	EKFC	Ma	Stevens	CKD-EPI	FAS
64	-24.2	-25.6	-26.1	-28.9	-24.0	-26.4	-23.3	-24.8	-26.2
20	-18.0	-7.9	-10.3	-8.8	-7.3	-6.3	-5.5	-5.6	-3.9
107	-13.0	-6.9	-7.2	-7.9	-8.6	-9.0	-8.2	-8.4	-8.7
42	-10.1	-8.6	-8.5	-9.7	-7.9	-6.9	-6.0	-6.6	-7.2
105	-9.7	-12.5	-12.4	-14.0	-14.4	-10.6	-9.2	-10.0	-11.6
52	-9.3	-0.8	-0.9	-0.9	-1.2	-2.6	-2.5	-2.3	-2.3
2	-9.1	-10.6	-13.1	-11.9	-14.0	-8.5	-7.3	-8.5	-7.5
74	-9.0	-9.2	-9.4	-9.9	-7.1	-6.5	-5.4	-6.2	-6.4
51	-8.3	-15.9	-15.5	-18.0	-5.4	-15.8	-13.5	-10.5	-10.0
55	-8.3	-17.5	-26.5	-19.8	-22.5	-13.6	-11.4	-14.3	-9.4
21	-7.5	-7.7	-9.1	-8.7	-7.6	-6.0	-5.1	-5.9	-5.2
98	-7.2	-3.6	-3.4	-4.5	-3.5	-3.9	-3.5	-3.4	-4.4
66	-6.7	-10.8	-11.1	-12.0	-9.3	-8.6	-7.3	-8.2	-8.4
53	-6.6	-14.7	-22.2	-16.5	-13.3	-15.4	-13.3	-15.9	-11.5
39	-6.5	-5.2	-6.9	-6.0	-7.2	-5.2	-4.5	-5.2	-4.3
25	-6.2	-6.4	-8.4	-7.3	-8.6	-6.3	-5.5	-6.2	-5.1
60	-6.2	-8.3	-13.2	-9.4	-8.4	-12.9	-11.6	-12.9	-9.6
94	-6.2	-9.8	-14.9	-11.0	-9.9	-8.8	-7.5	-9.3	-6.5
75	-5.9	4.7	2.4	4.9	1.1	3.6	2.9	2.0	3.0
129	-5.8	-25.6	-24.2	-28.4	-21.4	-16.3	-12.9	-13.6	-12.1
90	-5.6	-38.1	-30.3	-42.3	-25.9	-22.2	-17.4	-16.4	-14.2
28	-5.4	-7.4	-9.8	-8.4	-7.7	-8.0	-6.9	-8.1	-6.5
70	-5.4	-11.0	-13.3	-12.4	-14.1	-10.5	-9.0	-10.3	-9.4
114	-5.3	-8.6	-11.7	-9.7	-8.8	-6.6	-5.4	-7.0	-5.2
73	-5.1	-17.0	-18.9	-18.7	-14.7	-12.4	-10.2	-12.1	-11.3
125	-5.1	-11.5	-15.1	-13.1	-12.0	-8.6	-7.0	-9.1	-7.2
13	-5.0	0.7	0.5	0.7	0.4	0.1	0.0	0.1	0.4
38	-5.0	-8.5	-9.5	-9.3	-9.4	-9.2	-8.0	-8.7	-8.6
87	-5.0	-7.5	-7.4	-9.2	-7.1	-9.4	-8.2	-8.3	-9.6
76	-4.9	-3.5	-6.2	-4.0	-3.9	-7.9	-7.4	-5.3	-5.3
113	-4.8	-6.9	-8.9	-7.7	-8.9	0.3	0.9	-0.5	0.3
16	-4.4	-8.0	-10.8	-9.0	-8.2	-8.2	-7.1	-8.2	-6.4
26	-4.3	-7.9	-4.3	-9.0	-1.9	-12.5	-11.0	-8.4	-8.6
85	-4.2	-10.7	-12.9	-12.2	-10.8	-10.3	-8.8	-10.7	-9.8
84	-4.0	-1.6	-2.2	-1.8	-1.6	-5.8	-5.6	-5.5	-4.7
46	-3.8	-5.0	-4.8	-5.4	-4.2	-3.9	-3.3	-3.6	-4.0
103	-3.8	-8.6	-8.7	-9.4	-6.8	-8.0	-6.8	-7.4	-7.8
27	-3.6	-6.7	-9.8	-7.6	-7.1	-4.8	-4.0	-5.1	-3.0
93	-3.6	-11.8	-14.5	-13.0	-13.9	-11.2	-9.5	-11.0	-9.9
83	-3.4	-4.2	-3.4	-5.0	-5.6	-4.6	-4.0	-3.7	-3.5

24	-3.3	-6.3	-5.7	-7.6	-4.0	-3.4	-2.7	-3.2	-3.7
36	-3.3	-10.9	-11.8	-11.8	-12.2	-9.1	-7.8	-8.7	-8.7
119	-3.2	-0.4	-0.8	-0.6	-1.0	-0.5	-0.4	-0.6	-0.4
104	-2.9	-3.7	-4.3	-4.2	-5.0	-3.4	-2.9	-3.3	-3.1
4	-2.7	-3.9	-4.5	-4.5	-5.3	-3.6	-3.2	-3.5	-3.4
68	-2.7	-12.7	-17.6	-14.3	-13.1	-10.1	-8.5	-10.5	-7.6
29	-2.5	-7.7	-9.5	-8.7	-9.9	-5.9	-5.0	-5.9	-5.1
95	-2.5	-0.4	0.0	-0.7	-0.3	0.0	0.0	0.1	0.3
109	-2.5	3.2	4.3	3.4	3.3	-2.9	-3.0	-2.3	-3.6
132	-2.5	-7.4	-10.6	-8.5	-8.3	8.0	8.3	6.7	7.8
92	-2.3	-18.0	-20.2	-19.4	-18.6	-10.0	-7.9	-9.7	-8.8
124	-2.2	2.3	2.5	2.4	2.1	-1.9	-2.0	-1.5	-2.7
108	-2.1	-5.3	-6.2	-5.8	-4.6	-4.6	-3.8	-4.6	-4.4
116	-2.0	-10.2	-13.6	-11.6	-10.9	-9.8	-8.3	-10.0	-8.1
7	-1.9	-1.4	-3.7	-1.5	-1.9	-12.6	-12.0	-9.7	-9.5
120	-1.9	-20.0	-24.0	-22.6	-21.2	-18.4	-15.5	-17.2	-13.8
1	-1.8	-1.5	-2.1	-1.8	-2.6	-5.2	-4.9	-4.9	-4.3
34	-1.7	-4.6	-4.7	-5.1	-3.9	-4.6	-4.0	-4.3	-4.5
47	-1.7	-8.8	-9.2	-9.7	-9.6	-5.9	-4.9	-5.7	-5.7
59	-1.7	-59.7	-35.6	-67.3	-25.9	-39.7	-31.7	-25.6	-22.7
97	-1.7	-14.3	-15.7	-16.1	-13.4	-13.2	-11.4	-12.8	-12.6
23	-1.6	-9.9	-14.0	-11.0	-8.7	-8.1	-6.7	-8.4	-6.5
80	-1.5	-2.0	-2.8	-2.4	-2.4	-4.2	-3.8	-4.0	-3.4
56	-1.4	-4.1	-3.8	-4.6	-3.4	-2.9	-2.4	-2.7	-3.1
49	-1.2	-3.7	-5.9	-4.4	-4.2	-4.3	-3.8	-4.5	-3.2
63	-1.2	-2.0	-2.7	-2.3	-2.3	-3.3	-2.9	-3.2	-2.8
130	-1.0	-12.4	-18.1	-13.9	-11.4	-11.2	-9.7	-11.8	-8.3
30	-0.8	-4.8	-3.8	-5.1	-3.5	-3.4	-3.0	-3.1	-4.0
121	-0.7	-1.1	-1.2	-1.3	-1.2	0.7	0.7	0.4	0.5
37	-0.3	-4.2	-6.1	-5.0	-5.2	-2.0	-1.5	-2.3	-1.4
81	-0.3	2.7	4.1	3.1	2.1	-0.1	-0.6	0.6	0.5
115	-0.3	0.9	-0.1	1.0	0.1	-2.5	-2.6	-2.5	-1.8
118	-0.2	-3.6	-3.2	-4.4	-3.2	-4.1	-3.6	-3.5	-4.6
33	0.1	-10.8	-12.6	-11.7	-9.3	-16.8	-14.6	-10.8	-13.3
106	0.3	-6.8	-10.2	-7.8	-7.0	-5.5	-4.5	-5.8	-4.0
77	0.6	-11.0	-9.5	-12.4	-10.8	-10.3	-8.9	-7.5	-6.2
32	0.9	-8.9	-10.8	-9.6	-9.8	-14.5	-12.9	-13.4	-12.5
22	1.1	-2.2	-2.2	-0.6	-2.3	-1.1	-0.9	-1.2	-0.1
86	1.6	-1.2	-1.7	-1.5	-2.0	-3.2	-2.9	-3.0	-2.8
96	1.6	5.9	6.6	6.5	5.6	-6.7	-7.2	-5.7	-3.6
123	1.8	-17.3	-9.9	-19.5	-8.4	-11.5	-9.2	-7.0	-7.3
40	1.9	2.5	2.9	2.7	2.6	0.7	0.4	0.8	0.5
48	2.0	-7.2	-10.2	-8.4	-8.3	-8.0	-6.9	-8.2	-6.4
35	2.3	-2.1	-3.7	-2.4	-2.7	-1.4	-1.2	-1.5	-0.5
82	2.3	3.3	4.0	3.5	2.4	0.2	-0.2	0.3	-0.3
88	2.6	-8.5	-9.3	-9.5	-7.5	-6.1	-5.1	-5.9	-5.5

100	2.6	-2.3	-2.8	-2.7	-3.2	-2.6	-2.3	-2.5	-2.4
44	2.7	-13.8	-18.5	-15.6	-14.2	-13.4	-11.5	-13.6	-10.8
62	3.0	-7.9	-11.7	-9.0	-8.5	-7.4	-6.2	-7.8	-5.4
126	3.1	-0.2	0.7	-0.5	0.1	0.0	0.0	1.4	2.0
65	4.2	4.3	5.0	4.5	3.1	3.4	2.8	3.2	3.0
89	4.3	32.1	26.8	36.2	24.7	27.7	23.0	20.2	18.2
79	6.4	11.7	6.6	13.1	10.3	9.6	7.8	6.1	7.3
112	7.3	-0.4	-1.4	-0.4	-1.3	-10.1	-9.9	-5.9	-7.3
31	8.6	-9.7	-12.5	-10.7	-11.9	-7.1	-5.8	-7.2	-5.9
69	12.2	-4.7	-6.8	-5.5	-7.3	-2.7	-2.2	-3.1	-2.2
41	13.0	6.3	4.1	6.7	0.3	-2.0	-2.7	0.1	-1.8
**Counts**									
**≤-3**	43	69	71	69	69	72	69	73	72
**>-3 & <-1**	23	10	9	9	12	11	13	9	9
**≥-1 & <1**	11	7	6	7	6	9	10	9	10
**≥1**	20	11	11	12	10	5	5	6	6
**%**									
**≤-3**	30.7%	49.3%	50.7%	49.3%	49.3%	51.4%	49.3%	52.1%	51.4%
**>-3 & <-1**	16.4%	7.1%	6.4%	6.4%	8.6%	7.9%	9.3%	6.4%	6.4%
**≥-1 & <1**	7.9%	5.0%	4.3%	5.0%	4.3%	6.4%	7.1%	6.4%	7.1%
**≥1**	14.3%	7.9%	7.9%	8.6%	7.1%	3.6%	3.6%	4.3%	4.3%

End of Document.
